# Patient and caregiver perspectives of an early integrated systemic sclerosis palliative care clinic: a qualitative study

**DOI:** 10.1093/rap/rkaf098

**Published:** 2025-08-20

**Authors:** Carolyn Wicks, Julie McDonald, Laura Ross

**Affiliations:** Department of Palliative Care, St Vincent’s Hospital Melbourne, Fitzroy, VIC, Australia; Department of Palliative Care, St Vincent’s Hospital Melbourne, Fitzroy, VIC, Australia; Department of Respiratory Medicine, St Vincent’s Hospital Melbourne, Fitzroy, VIC, Australia; Department of Medicine, University of Melbourne, Parkville, VIC, Australia; Department of Medicine, University of Melbourne, Parkville, VIC, Australia; Department of Rheumatology, St Vincent’s Hospital Melbourne, Fitzroy, VIC, Australia

**Keywords:** systemic sclerosis, palliative care, symptom control, models of care

## Abstract

**Objectives:**

Individuals with systemic sclerosis (SSc) and their caregivers have unmet palliative care needs, yet the role of palliative medicine in SSc is unclear. In this study we aimed to explore patient and caregiver perspectives of a newly developed early, integrated SSc-specific palliative care clinic.

**Methods:**

All patients and caregivers who attended the SSc Palliative Care Clinic within the first 6 months of its implementation were invited to participate in a semi-structured phone interview. Interviews were audio-recorded, transcribed and analysed using reflexive thematic analysis.

**Results:**

Six patient interviews, three caregiver interviews and three patient–caregiver dyad interviews were performed. The SSc Palliative Care Clinic was valued and accepted by patients and caregivers. The experience of living with and caring for SSc was described in all interviews. Four further themes were identified, describing the experience of attending the SSc Palliative Care Clinic: the valued integrated structure of the clinic, including the value of interdisciplinary care; respectful communication style of the physician that patients found non-judgemental, supportive and empathetic; the dichotomy of receiving palliative care that included the relief of discussing symptom burden and hope arising from active symptom management; and diverging views of future care discussions.

**Conclusion:**

The SSc Palliative Care Clinic was both accepted and valued by patients and caregivers. Findings highlighted the need for palliative care delivery to be sensitive and person-centred, with such care being observed to foster hope and optimism for both patients and their caregivers.

Key messages

- Systemic sclerosis patients and caregivers found patient-centred and disease-specific palliative care acceptable and highly valuable.
- Integrated palliative care for systemic sclerosis patients may improve symptom control and quality of life.
- Potential barriers to care include misconceptions of the scope of care provided by palliative care.

## Introduction

Systemic sclerosis (SSc) is an incurable autoimmune disease that causes organ damage across multiple organ systems. This multisystem organ involvement means the symptom burden of SSc is high and is associated with significant physical disability [1, 2].

The World Health Organization recognises palliative care as a distinct specialty that prevents and relieves suffering by addressing the physical, psychological, social and spiritual challenges associated with disease. The role of palliative care is recognised in the management of heart failure and chronic lung and neurological diseases, which are all associated with an intractable illness course and large symptom and caregiver burden [3–5]. Integration of palliative care as one aspect of holistic ‘active’ chronic disease management is now part of standard care procedures for many chronic diseases [6–8]. Hospital-based palliative care can improve quality of life and symptom control, as well as caregiver satisfaction [9]. Integrated palliative care for advanced lung disease has resulted in fewer hospital admissions and healthcare cost savings [10].

Considering the benefits of palliative care in the management of other diseases, we considered the need for palliative care in SSc [1, 11]. Three-quarters of SSc patients may have specialist palliative care needs, such as for management of severe fatigue, breathlessness, pain, depression, anxiety and gastrointestinal symptoms [1]. In response, we implemented an integrated SSc Palliative Care Clinic, embedded within the rheumatologist-led SSc Clinic. In this qualitative study we sought to evaluate the patient and caregiver experience of the clinic. We aimed to assess whether patients and their caregivers were satisfied with their experience at the SSc Palliative Care Clinic, assess the appropriateness of clinic interventions and identify any gaps in the clinical service provided to patients.

## Methods

### Study design

This qualitative study used semi-structured interviews and a reflexive approach to thematic analysis [12]. This approach was selected to permit the identification and interpretation of patterns and themes within the interviews, providing a detailed and nuanced understanding of experiences and perceptions of the newly developed clinical model. An open analytical approach was intentionally selected to enable identification of patient and caregiver insights independent of the research team’s preconceptions or hypotheses [12, 13].

### Study setting

The SSc Palliative Care Clinic is a physician-led ambulatory care clinic in an academic tertiary hospital staffed by a physician with specialty training in both respiratory and palliative medicine. Patients were referred to the SSc Palliative Care Clinic if they had high symptom burden, frequent hospital admissions, decreased function and increased reliance on caregivers, were being considered for lung or autologous stem cell transplantation, patient request for future care discussion or physician-perceived poor prognosis. All patients were informed of the aims of the SSc Palliative Care Clinic at the time of referral and all provided verbal consent to clinic referral.

**Figure 1.**
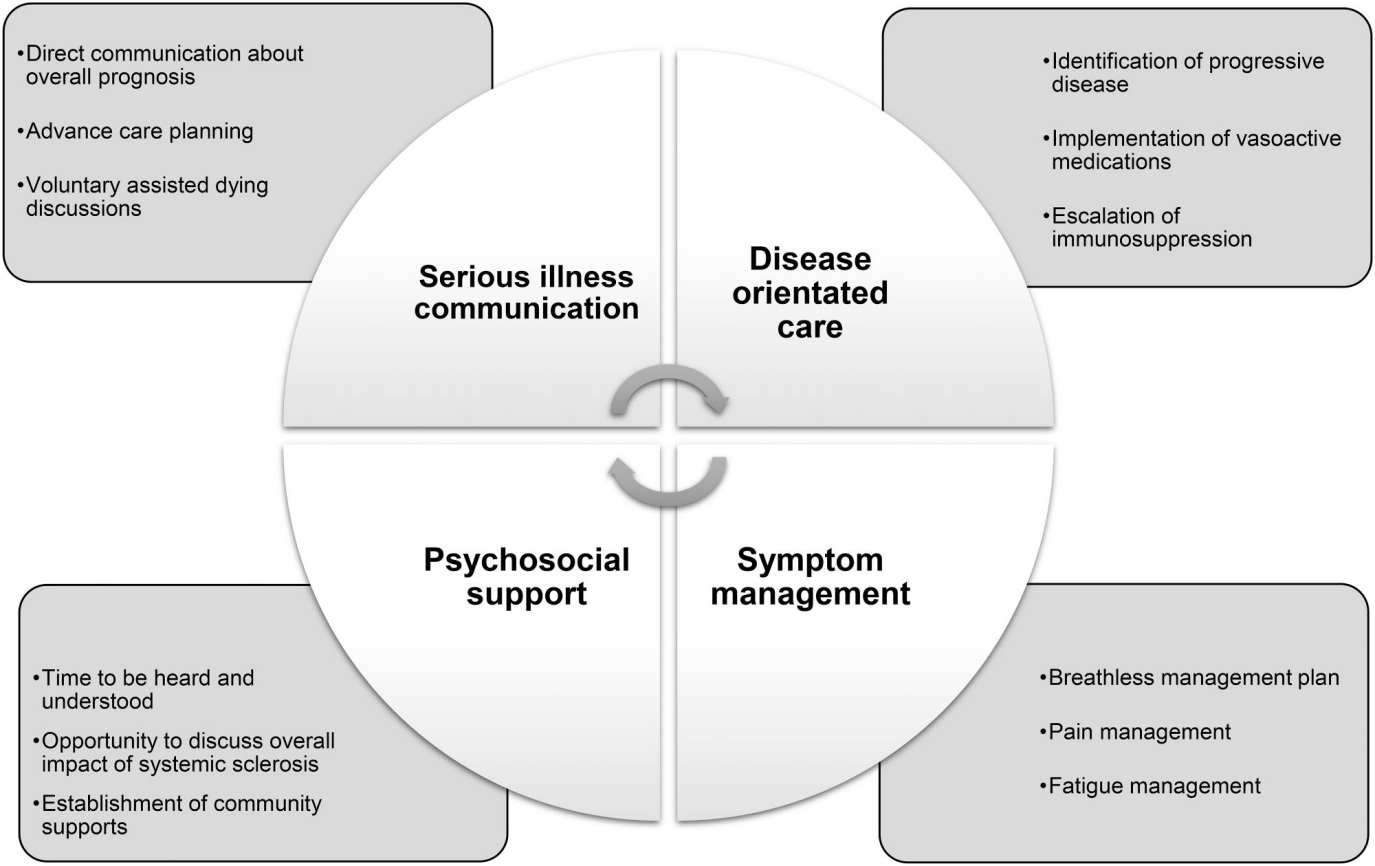
Pillars of integrated palliative care with examples of care delivered at the SSc Palliative Care Clinic

The SSc Palliative Care Clinic was co-located with a specialist SSc clinic. The model of care has been described elsewhere [14]. No formal guidelines exist for the implementation of palliative care for individuals with SSc, therefore care was focused on delivering treatment that addressed the pillars of palliative care that have been identified in the management of other chronic diseases [4, 5, 10, 15–17]. These pillars focus on the delivery of patient-centred care that aims to improve both patient and caregiver quality of life by integrating active symptom management and psychosocial support with disease-oriented care (Fig. 1).

### Study participants

All patients and caregivers who attended at least one SSc Palliative Care Clinic appointment between July 2023 and January 2024 were invited to participate. All patients were contacted 2–8 weeks after their appointment. Caregivers were nominated by the patients and were only contacted if they attended the SSc Palliative Care Clinic appointment with the patient. If participants were not initially contactable, the interviewer tried twice more on separate occasions. If participants remained uncontactable after three attempts, they were classified as uncontactable.

### Data collection

A semi-structured interview guide was developed, adapting questions that were applied to an evaluation of a respiratory palliative care service (Supplementary Index 1, available at *Rheumatology Advances in Practice* online) [17]. All participants were interviewed by telephone by a study investigator not involved in the delivery of care at the SSc Palliative Care Clinic. All interviews were conducted in English, audio recorded and transcribed verbatim. All transcripts were anonymised and reviewed for accuracy prior to analysis. Field notes, including reflexive practices, were taken during the interviews. Demographic information and medical history were extracted from the medical record.

### Data analysis

Reflexive thematic coding was used to identify, analyse and report themes within the data using a six-phase process: familiarisation, coding, theme development, review, definition and reporting of themes [12, 18]. All study investigators were medical doctors with experience in qualitative research methods. Two investigators were not directly involved in the SSc Palliative Care Clinic. A third investigator was a clinician from the SSc Palliative Care Clinic. All investigators have experience in the assessment and clinical management of SSc and have clinical and research interests in the management of complex chronic disease.

All transcripts were read by all study investigators to familiarise themselves with the text. Each investigator independently coded all interview transcripts to identify important aspects of the data. Regular consensus meetings were held with all three investigators present to minimise bias and determine themes. Study investigators reflected on their interpretation of the transcripts and identification of codes and themes and discussed any biases that may have arisen from their involvement in the care of participants and involvement in study interviews or coding of the data. Themes and their definitions were reviewed iteratively to develop clear theme definitions, ascertain their relevance to the research aims and ensure quote context was maintained.

This study was approved by the Human Research Ethics Committee at St Vincent’s Hospital Melbourne (2023/PID00241). All participants provided verbal consent prior to the collection of any study data.

## Results

Sixteen patients attended 21 appointments during the first 6 months of the SSc Palliative Care Clinic. Eleven patients (69%) participated in this study. Five patients did not participate in this study because they did not speak English (*n* = 2), were uncontactable (*n* = 2) or died prior to follow-up (*n* = 1).

Twelve interviews were completed relating to care delivered to 11 patients. Six interviews were performed with the patient, three interviews with caregivers and three interviews with the patient and their caregiver together. Patient age ranged from 19 to 76 years, 64% were female and 55% had diffuse SSc. The median time from SSc diagnosis to interview was 9 years (range 0–38). One-third of patients ages 18–65 years worked at least part time. The median Australia-modified Karnofsky Performance Status [19] was 60 (range 40–70), signalling ‘able to care for most needs but requiring some assistance with activities of daily living’. Most patients were referred to the SSc Palliative Care Clinic for symptom management (82%), while 54% were referred for future care discussion. Most patients (82%) identified a caregiver, many of whom (89%) lived with the patient.

We identified six themes within the dataset. Two themes described the lived experience of SSc from the patients’ and caregivers’ perspective. Another four themes reflected the participants’ experiences attending the palliative care clinic: the value of integrated care, communication style of the physician, the dichotomy of palliative care and diverging views of future care discussions.

### Living with and caring for SSc

#### The daily impact of SSc

Despite this study including no specific questions pertaining to the overall experience of living with SSc, the profound daily impact of SSc and the attendant uncertainty about the future and overall prognosis was consistently described (Table 1). Patients and caregivers described the existential nature of their grief about living with SSc and the associated loss of independence. Participants expressed distress about limitations of mobility, difficulties with activities of daily living such as toileting and walking and their heavy reliance upon caregivers. The unpredictability of the disease course of SSc was highlighted. Patients described being equally surprised by periods of unexpected improvement:

**Table 1.** Living with and caring for SSc.

Participant	Quotation
Daily impact of SSc	
Patient E	I laugh around everything, I laugh, I try not to see the sad bad sides, I try and get around everything…(and) plod along and do the best we can.
Patient H	I don’t want to think about the future…it’s going to happen anyway. If I think about it, it makes me depressed…It’s dramatically different to what I was doing six months ago, but I’m still getting out there and trying to move. I walk like an old person now. I walk really slow and my arms are just set in a certain position. Basically I can’t even wipe my own [bottom] now, if I have a [bowel action] I have to have a shower.
Patient L	Getting systemic sclerosis isn’t a positive experience. And I’m an A-type personality so I haven’t found my lack of movement and restriction easy to deal with.
Patient N	There are days where I don’t feel like doing this, there are plenty of other times I’m fine, so I just pace myself…I think you tend to learn to live with some of the short comings that you have because they don’t all appear at once, they just happen and you make adjustments accordingly.
Caregiver A	I sort of know that they’ve got a weird illness that’s not going to be cured any time…A lot of the time, like they are at the moment, they’re well, out in the garden for two hours this morning and other times they get unwell. Last month, for a couple of weeks they were sliding down hill.
The caregiving dilemma—gratitude and burden	
Patient L	[Caregiver] is the sort of person that gets involved. They’re always researching let me tell you; was it Russia or Germany last week that had some trial? It goes on and on and on.
Patient I	My main concern is that I don’t want my partner or my kids to be the ones to be pulling the plug. I haven’t had a proper conversation with anyone else about that. I don’t want them…I feel like that is a burden.
Caregiver E and Patient E	I go to every appointment with them, I probably understand more than they do. [Patient E]: To me it’s a blur. [Caregiver] is in full control of my brain cells, without them I wouldn’t have a clue.
Caregiver V	I’m more trying to make their life as comfortable as possible for as long as we’ve got.

It surprises me all the time, I didn’t think I would recover as well as I did when I aspirated and got pneumonia. (Patient Y)

as by periods of significant decline:

I’ve cruised along with very little impact on my life…and suddenly the pulmonary hypertension has come and I’m very unwell and still horrified by it. (Patient F)

Participants made frequent references to the resilience required to manage living with SSc and the insidious nature of their increasing symptom burden and physical disability.

#### The caregiving dilemma—gratitude and burden

The role and contribution of caregivers was universally appreciated by patients who identified a caregiver (Table 1). The primary role of caregiving was maintaining patient well-being with numerous other responsibilities described, including assistance with personal and domestic activities of daily living, medication administration, advocating for and coordinating care, attending medical appointments and synthesising health information. Patients frequently described their dependency on caregivers, and the burden of caregiving was acknowledged by patients. One caregiver described SSc as a ‘hell of a ride’ (Caregiver O), and the all-encompassing nature of caregiving was described by both patients and caregivers. Patients expressed guilt about their care needs:

I sort of feel guilty I have [a caregiver] all the time. They need to have their own life, not look after me’ (Patient E).

Caregivers frequently expressed a willingness and preference to be involved in clinical decision-making, describing that ‘someone pull me aside and say hey look, this is what’s going on’ (Caregiver O) in the event of any important clinical change.

### Experience of SSc Palliative Care Clinic

Attendance at the SSc Palliative Care Clinic was the first experience of palliative care for most patients. Patients reported their attendance was a positive experience and that they were supported and actively helped.

[Physician] has got me to where I am at now…despite being very sick, I am at a good place so I figure it must be working. (Patient Y)

Participants expressed optimism they had further treatment options to better manage their symptoms:

[The review] gives me confidence if you like, as you always think is there another solution. (Patient L)

When patients and caregivers were asked what type of care they received, they described a range of care tasks. General well-being and social support networks were frequently discussed. Patients recalled being asked about both informal and formal support services at home and whether these met their perceived care needs. Patients and caregivers appreciated the time taken to explain investigation results and assistance to understand the role and benefits of various treatment strategies. Symptom management plans that included pharmaceutical as well as lifestyle and behavioural strategies to optimise function and prevent deterioration were implemented. Patients and caregivers frequently reported that symptom management plans helped them feel more confident in the daily management of their disease; they were described as ‘helpful, definitely’ (Caregiver O) and that they helped ‘[patient] understand more, having the steps (to manage severe breathlessness)’ (Caregiver E) and to feel more ‘motivated…they gave me a good explanation of the importance of the exercise to get my lungs as best I can’ (Patient F). Caregivers valued being included in the consultation, reflecting ‘[Physician] talked to me equally’ (Caregiver E). Patients frequently documented their advance care plans and received assistance with the appointment of powers of attorney.

#### Respectful communication style

Patients reported the palliative care physician was supportive, encouraging and empathetic (Table 2). They described being well listened to and that their condition and concerns had been understood. Patients and caregivers highly valued the time and opportunity to describe their unique experiences. Participants felt their preferences and choices regarding topics of conversation and care needs were respected. Difficult conversations were approached with sensitivity and respect and if requested, discontinued:

**Table 2.** Respectful and positive communication at SSc Palliative Care Clinic.

Participant	Quotation
Patient I	We know what’s coming, me and my [partner], we know what to expect in the future and we were more than happy when [physician] was open and straightforward telling us what the things like that are and how we can make it a lot easier. I was a bit reluctant to take the oxygen but [physician] was requesting me to get the [oxygen saturations] up, and all those things will help me. Before the oxygen I was actually struggling, she said with the oxygen I would probably get more things done, which can help me more.
Patient L	[Physician] sort of makes me feel good, as they’re a very positive person. It’s been a very positive experience for me. My initial consultation she sorted out my medications, my drugs, so far it’s been a good call, what she sorted…and I’ve done my end of life (advance care directive), [physician] helped me sort that out—she sent the copies out to look at, fill out and sign, and return to the hospital…For me it’s been positive and supportive. What I need for my personality type.
Patient Y	We talked about voluntary assisted dying as that was playing on my mind a lot, especially after hearing about the possibility of me dying from a bowel obstruction. That’s been my greatest fear…I didn’t feel [physician] disrespected my decisions, or questioned my thought process, or intellect in how I understood how voluntary assisted dying worked.
Caregiver R	I was very happy with [physician], she knew what she was about as far as I was concerned. She was very switched on and covered everything properly, professionally.

They started talking about family support and friends but then I shut [physician] down…they did exactly what I asked them to. (Patient H)

Many patients appreciated direct communication about their illness and prognosis:

I would rather someone who is dealing with it, rather than just giving off the vibe. They said it as it was. (Patient V)

The importance of sensitive delivery of questions and advice pertaining to difficult topics such as increasing care needs and future care plans was highlighted. Such topics were identified as ‘confronting and hard’ (Caregiver A), but patients reported the physician was

…gentle with the responses, not that brutal in the delivery…we need to know the truth but the delivery is also important. (Patient Y)

#### The integrated structure of the SSc Palliative Care Clinic

Patients and caregivers universally described SSc as a complex condition, and they valued coordinated and specialist care from an interdisciplinary team. They valued physicians who understood the complexity of their condition, as participants described difficulty finding healthcare professionals who understood the disease. Some participants remained uncertain as to the role of the palliative care physician, describing being ‘not completely sure’ (Caregiver O) or being uncertain as to why care was delivered by a palliative care physician: ‘Why did I see palliative care?’ (Patient H). Some patients were confused by the complexity of their care team in general, never being sure ‘who do I ring?’ (Patient L). Patients and caregivers did not describe major gaps in the care they had received.

Many patients and caregivers valued the ‘overall coordination’ that took place ‘in house’ (Caregiver L) at the integrated clinic. Patients and caregivers appreciated that the clinic was co-located with the rheumatology-led SSc clinic so they could ‘get it done on one day’ (Patient E). Patients appreciated the clear communication from the SSc Palliative Care Clinic to their general practitioner and community palliative care services.

#### The dichotomy of receiving SSc palliative care

Some patients and caregivers expressed distress at being referred to palliative care and found the label palliative care confronting. However, almost universally, patients who expressed initial distress or hesitation about their referral also expressed relief and gratitude for the opportunity to discuss symptom burden and management and future care planning (Table 3). Patients described not previously considering palliative care relevant to themselves. One patient described being ‘devastated’ at being referred to the palliative care clinic:

**Table 3.** The dichotomy of SSc palliative care.

Participant	Quotation
Patient E	I am sort of expecting but I don’t want to hear it because I still think I’m young and have a long time left. Whenever I hear the words ‘palliative care’ it’s not something that’s good for me, as I lost my wife with palliative care. I don’t like hearing those words or want to go down that track myself yet, though I accept it has to be done, yes.
Patient F	I have a [family member] who is a nurse and I did tell them and that I was devastated by the whole thing and they said don’t look at it that way, that [palliative care] is actually quite a positive thing…It was more ignorance on my part I think.
Patient I	I was reluctant to do palliative care, but I think it was the right time and the right call.

When someone says I’m with palliative care, you can imagine how upsetting that is… (But) [physician] was very encouraging to me, I was very low, so it was a very positive experience for me. I really felt like I could get some support. (Patient F)

Some patients described their own internal conflict about the involvement of palliative care in their management but were open to education about the role of palliative care in chronic illness:

…(it was) stressed that palliative care is anything to make my life more easy, not in a palliative home, but in every-day life. (Patient N)

#### The divergence in acceptance of future care discussion

There were diverging views regarding the acceptability of serious illness discussion, prognostication and advance care planning. While some patients and caregivers described a pragmatic acceptance of the future and a preference for being prepared, others described a fearful denial of the future (Table 4). Those who accepted future care discussions spoke of how the discussions positively impacted them:

**Table 4.** Diverging views about future care discussions.

Participant	Quotation
Patient H	It’s not hard to talk about, I just don’t want to know about it. Once I come up to it, I’ll deal with it. I’m still living. I don’t want to think about the future.
Patient L	It didn’t cause me anxiety as I’m not that sort of person.
Patient N	It would be better if [my partner] was with me, I probably should have a meeting, [Partner] needs to understand. I said to them not long ago that I’m actually not going to get any better than I am now, I think they think it’s something I am going to get over. It’s hard for anyone to envisage deterioration.
Patient R	I had it at the back of my mind I guess and I was prepared. I guess I am one of those people who sort of think in advance of what I want and how I want things to be. I guess, I don’t put my head in the sand.
Patient V	(speaking to caregiver): We have an advance care plan, haven’t we? We’ve shown the kids but they don’t want to see it. I can understand that.
Caregiver R	We had spoken to [physician] earlier on the phone about the advance care plan and making sure everything was filled out, medical power of attorney and all the rest of it. When someone who knows what they are talking about, you prick your ears and think about the future and where it could lead to.

From my first introduction to [physician] I pretty much organised everything—I wouldn’t be where I am now if I hadn’t had those conversations…everything is thought through, planned…I have organised everything, down to my funeral. (Patient Y)

Some patients were able to name reasons for avoiding any discussion of their potential future care needs. Patient E described not wanting to ‘hear it because I still think I’m young and have a long time left’. However, Caregiver E in the same interview was able to describe their desire for ‘plans…as an emergency back-up’ in recognition that ‘[Patient E] is hopeful, I’m probably not as optimistic’. A fear of being denied future treatment was expressed by one caregiver:

They think if they commit to any plan not to resuscitate them, [doctors] are not going to help them at the hospital. It’s like you’re giving them permission to die and not treat them. (Caregiver A)

Some patients identified their own preparedness to discuss future care needs and ‘think about some different options’ but their family was not ‘wanting to hear about it’ (Patient N). Patient R expressed comfort with discussing the future but described their caregiver’s reticence: ‘They don’t like to talk about that sort of thing, but I always have’.

## Discussion

In a disease where there is high unmet symptom burden and a frequently intractable illness course [1, 2], we have demonstrated palliative care interventions delivered in a personalised, disease-specific manner are both valued and well accepted by SSc patients and their caregivers. Care tasks completed in the palliative care clinic included symptom management, psychosocial support and serious illness communication and were integrated with the delivery of disease-specific treatment. Almost universally, participants appreciated having the opportunity to discuss their experience of SSc and symptom burden and develop both pharmacological and non-pharmacological management plans to better manage their symptoms. Implementation of palliative care that is integrated and delivered hand-in-hand with disease-specific care may offer the opportunity for improved patient outcomes and quality of life. The role, and importance to patients, of multidisciplinary care in the long-term management of SSc is recognised [20, 21]. Patients seek clarity about treatment decisions and future care planning [22]. The recently published British Society of Rheumatology SSc treatment guidelines referenced the potential for palliative care to be an adjunctive treatment to improve quality of life in those patients with severe SSc or for those nearing the end of life [23]. Palliative care has the potential to offer psychosocial and spiritual support as well as specialised symptom management and assistance with the setting of future treatment goals, in addition to the care that can already be provided by multidisciplinary care teams for those with severe SSc.

Participants identified the physician’s communication style and provision of symptom management plans as important aspects of gaining symptom mastery and instilling optimism. Hope in the face of chronic, intractable illness is well described in oncology settings and is associated with improved patient well-being and quality of life [24]. Provision of hope to patients with an incurable disease is an important aspect of early, integrated palliative care. Early palliative care can equip patients with skills to cope, help patients live fully despite their illness, prepare for the future in a gradual process and remain sufficiently well to engage with other aspects of their care [25]. Previous data examining patients’ preferences for SSc care have highlighted the importance of clear communication both between physicians and patients and between care providers and those living with SSc [20, 26]. Patients with SSc have described a desire for improved non-pharmacological management strategies to optimise symptom management [20], which is consistent with our findings that symptom management plans developed with the palliative care physician were welcome and valued by patients.

We elected to co-locate the SSc Palliative Care Clinic alongside the rheumatology-led SSc Clinic to facilitate communication between team members and minimise healthcare burden on patients. SSc patients reported the benefits of direct and clear exchange of information between specialists and the convenience of attending both clinics on the same day. Embedded or co-located palliative care clinics are thought to offer enhanced collaboration, earlier palliative care involvement and real-time combined therapeutic decision-making [27]. The problems arising from long travel times to access specialised healthcare and the frequent need for disease-specific symptom management further support the co-location of palliative care clinics for SSc patients [1, 2, 14, 26]. While we were unable to identify any unmet care needs in this study, the focus of this study was restricted to patients’ and caregivers’ experiences of clinic attendance, which may not have captured other unmet care needs. Unmet care needs should be explored further in future studies that aim to develop novel models of care for SSc.

A misconception of palliative care and conflation of palliative care with end-of-life and ‘giving up’ have been identified as barriers to the provision of early palliative care [3, 28, 29]. Some participants in this study identified this association and found receiving care labelled as palliative challenging. These participants expressed concern they may now be receiving end-of-life care rather than active SSc treatment. Apprehension or fear at referral to palliative care has been similarly reported in patients with interstitial lung disease [30]. However, early integrated palliative care aims to foster therapeutic relationships that develop over months to years, permitting continuity of care and relationships that facilitate living well with serious illness [25]. It was notable in this study that some patients were able to reflect on a change in their attitude towards palliative care as a result of education from their physician or family members and their direct experiences at the SSc Palliative Care Clinic.

We observed a divergence in preference for future care discussion and advance care planning. Serious illness communication is a structured communication strategy that focusses on a patient’s current illness experience and includes an assessment of illness knowledge and patient preference for further information. It acknowledges and responds to emotions, as well as explores patient goals and priorities and makes medical recommendations when required [31]. Serious illness communication and any advance care planning are dynamic phenomena that should occur as part of the continuum of care provided across the life course of an individual patient [32]. In other non-malignant diseases, patients have expressed a preference for early introduction of the concepts of advance care planning, but have voiced a preference for physicians to initiate such discussions [33–35]. Young age and uncertain disease trajectory have both been identified as barriers to the initiation of such discussions [33, 34] and the optimal timing and frequency of such communication strategies are uncertain.

This study is not without limitations, including the small sample size, and it included only individuals who had been referred to the SSc Palliative Care Clinic because of an identified unmet care need. The generalisability of these findings may be limited, as the attitudes towards palliative care of the broader SSc patient community may not be reflected in this study. Participants were interviewed up to 8 weeks after their clinic consultation. The delay in data collection may have affected participants’ recall of their consultation. However, time elapsed between the consultation and interview may have permitted reflection on the effects of clinic attendance that may not have been captured in an immediate interview. Two investigators had therapeutic relationships with study participants, one of whom was involved in the delivery of care in the SSc Palliative Care Clinic. Investigator subjectivity informing data analysis has been considered a strength of reflexive thematic analysis, and the subjectivity arising from clinical experience and qualifications has informed the analysis of this dataset [12]. However, to address the potential biases that may have arisen from the interpretation of data by an individual investigator, all transcripts were read by all three investigators. Reflexive practices including discussion of personal biases were embedded within consensus meetings to address these potential biases in data analysis.

This study is the first documentation of SSc patient and caregiver perceptions and experiences of palliative care. Patient care focused on the quality of life of patients and their caregivers and communication with patients about the uncertainty of their illness and their future. We have documented that this care model is acceptable, positively received and contributes to a sense of optimism and improved symptom mastery by patients. Future work is required to ascertain the perceptions, and palliative care needs, of the larger SSc patient and caregiver community. More data are required to define an optimal model of integrated palliative care and formally test the efficacy of palliative care interventions in SSc. However, our preliminary results suggest there is a positive role for early, integrated palliative care to improve symptom control for individuals living with SSc.

## Supplementary Material

rkaf098_Supplementary_Data

## Data Availability

The data underlying this article cannot be shared publicly due to the protection of the privacy of individuals who participated in the study. The data will be shared upon reasonable request to the corresponding author.
