# Supplementary material for: Patient and caregiver perspectives of an early integrated systemic sclerosis palliative care clinic: a qualitative study
Source: Rheumatol Adv Pract. 2025 Aug 20;9(4):rkaf098. doi: 10.1093/rap/rkaf098 (PMC12448292; doi:10.1093/rap/rkaf098)
Supplement: rkaf098_Supplementary_Data [file rkaf098_supplementary_data.zip › 25-036 Supplementary Table S1.docx]

*Supplementary Table S1: Interview guide*

| - What did you talk about at the SSc Palliative Care Clinic? |
| --- |
| - Did you feel the doctor understood your health concerns? |
| - Were you given a plan to manage your symptoms? |
| - When the doctor saw you, did they change any medications or hospital appointments? |
| - Did you have any discussion about your care in the future?   - If yes, did you record an advance care plan during your consultation? |
| - Were you satisfied with the service you received at the SSc Palliative Care Clinic? |

*Abbreviations:* SSc: systemic sclerosis
